# Application value of blood metagenomic next-generation sequencing in patients with connective tissue diseases

**DOI:** 10.3389/fimmu.2022.939057

**Published:** 2022-08-01

**Authors:** Rui Su, Huanhuan Yan, Na Li, Tingting Ding, Baochen Li, Yuhuan Xie, Chong Gao, Xiaofeng Li, Caihong Wang

**Affiliations:** ^1^ Department of Rheumatology, the Second Hospital of Shanxi Medical University, Taiyuan, China; ^2^ Pathology, Joint Program in Transfusion Medicine, Brigham and Women’s Hospital/Children’s Hospital Boston, Harvard Medical School, Boston, MA, United States

**Keywords:** connective tissue diseases, metagenomic next-generation sequencing, virus, infection, EBV - Epstein-Barr Virus, CMV (citomegalovirus)

## Abstract

**Objective:**

This study aimed to analyze the application value of blood metagenomic next-generation sequencing (mNGS) in patients with connective tissue diseases (CTDs) to provide a reference for infection diagnosis and guidance for treatment.

**Methods:**

A total of 126 CTD patients with suspected infections who were hospitalized in the Department of Rheumatology, the Second Hospital of Shanxi Medical University from January 2020 to December 2021 were enrolled in this study. We retrospectively reviewed the results of mNGS and conventional diagnostic tests (CDTs).

**Results:**

Systemic lupus erythematosus (SLE) and polymyositis/dermatomyositis (DM/PM) had the highest incidence of infections. The positive pathogen detection rates of mNGS were higher than those of CDT. The virus infections are the most common type in CTD patients with single or mixed infection, especially Human gammaherpesvirus 4 (EBV), Human betaherpesvirus 5 (CMV), and Human alphaherpesvirus 1. The incidence of prokaryote and eukaryote infections is secondary to viruses. Bloodstream infections of rare pathogens such as *Pneumocystis jirovecii* should be of concern. Meanwhile, the most common mixed infection was bacterial–virus coinfection.

**Conclusion:**

mNGS has incremental application value in patients with CTD suspected of co-infection. It has a high sensitivity, and a wide detection range for microorganisms in CTD patients. Furthermore, the high incidence of opportunistic virus infections in CTD patients should be of sufficient concern.

## Introduction

Connective tissue diseases (CTDs) are a group of diseases with a variety of clinical manifestations caused by immune-mediated chronic inflammation, influencing various connective tissues of the body (1). The pathogenesis is complex, involving many factors such as genes, environments, and immune factors. The fatality rate of CTD such as systemic lupus erythematosus and dermatomyositis is high and there is no current cure. The main therapeutic strategies are glucocorticoids and immunosuppressive drugs, but the effects are not satisfactory and the side effects are obvious, leading to the increase of infection (2–4). Autoimmune disorders and treatments give rise to susceptibility for infections in CTD, it is reported that the risk of opportunistic infection was highest for dermatomyositis and polymyositis/dermatomyositis (DM/PM), followed by systemic lupus erythematosus (SLE) (2, 5). Infections have become important causes of morbidity and mortality in CTD patients. Effective anti-infective treatments at an appropriate time is essential to reduce infection mortality and improve disease response rates. Thus, accurate and early identification of pathogens and targeted anti-infective treatment are crucial to the prognosis of such patients. However, because of impaired immune function of CTD patients, there are many problems such as difficult pathogen diagnosis and complicated infection types. Despite various tests available for infections, rapid and accurate diagnosis and identification of causative pathogens continue to face great challenges.

Metagenomic next-generation sequencing (mNGS) has emerged as an effective and universal pathogen detection method for infection diagnostics in recent years (6–9). Compared with conventional microbiological tests, mNGS has the advantages of more diverse detection samples including bronchoalveolar lavage, sputum, puncture fluid, and urine; a wide range of detectable pathogens including viruses, bacteria, and fungi; and shorter analysis time. Based on the application of mNGS, we can identify pathogens earlier, including those that are limited by conventional microbiological tests in the clinical diagnosis and treatment of CTD. mNGS can be used to extensively analyze the microbiome of clinical samples.

However, there are few studies on the application of the blood mNGS method in CTD patients with suspected infections. Therefore, we analyzed the application value of blood mNGS in patients with CTD to provide a reference for the diagnosis and guidance for treatment.

## Materials and methods

### Study design and participants

This is a retrospective study that analyzed 126 CTD patients with suspected infections admitted to The Second Hospital of Shanxi Medical University from January 2020 to December 2021. All patients with CTD were diagnosed according to relevant diagnostic criteria, including 34 SLE, 24 DM/PM, 19 rheumatoid arthritis (RA), 10 undifferentiated connective tissue disease (UCTD), 16 Sjogren syndrome (SS), 5 mixed connective tissue disease (MCTD), 5 ANCA-associated systemic vasculitis (AAV), 5 adult onset Still’s disease (AOSD), 2 Behcet’s disease (BD), 2 primary biliary cholangitis (PBC), 2 takayasu arteritis (TA), 1 systemic sclerosis (SSC), and 1 retroperitoneal fibrosis (RPF). The inclusion criteria were as follows: (1) highly suspected infection adult patients with diagnosed CTD; (2) complete medical record. The exclusion criterion was incomplete medical record. The above samples were tested for conventional microbiological tests and mNGS. This study is a retrospective study based on the examination results of previous clinical diagnosis and treatment. The Second Hospital of Shanxi Medical University ethics committee granted our application for an exemption from informed consent.

### Laboratory data

Data on the clinical and serological parameters of these patients were collected retrospectively, including blood routine, erythrocyte sedimentation rate (ESR), and C-reactive protein (CRP). Conventional diagnostic tests (CDTs) include routine culture of microbes including aerobic and anaerobic bacteria, fungi, and acid-fast bacilli; determination of Epstein–Barr virus (EBV) and cytomegalovirus (CMV)-DNA; mycoplasma and influenza virus serological tests; tuberculosis tests (T-SPOT.TB); and fungal assays (Aspergillus galactomannan and fungal beta-d-glucan). The above blood tests were performed in the laboratory of our hospital.

### Metagenomic next-generation sequencing and analysis

#### Nucleic acid extraction

Enough whole blood (adults 5–10 ml) was collected in Cell-Free DNA BCT STRECK. and then stored or shipped between 6 and 35°C to Hugobiotech Co., Ltd. (Beijing, China) to perform mNGS detection immediately. The DNA was extracted and purified from samples using QlAamp DNA Micro Kit (50) #56304 according to the manufacturer’s instruction. DNA concentration and quality were checked through Qubit and agarose gel electrophoresis.

#### Library generation and sequencing

The DNA libraries were constructed using the QIAseq™ Ultralow Input Library Kit. The concentration and quality of libraries were checked using Qubit and agarose gel electrophoresis. Qualified libraries with different barcode labeling were pooled together, and then sequenced on an Illumina Nextseq platform.

#### Bioinformation pipeline

After obtaining the sequencing data, high-quality data were generated after filtering out adapter, low-quality, low-complexity, and shorter reads. Next, remove human reads by mapping reads to human reference genome using SNAP software. The remaining data were aligned to the microbial genome database using Burrows-Wheeler Alignment. The database collected microbial genomes from NCBI (ftp://ncbi.nlm.nih.gov/genomes/). It contains more than 20,000 microorganisms, including 11,910 bacteria, 7,103 viruses, 1,046 fungi, and 305 parasites. Finally, obtain the microbial compositions of the sample.

### Statistical analysis

SPSS 20.0 software was used for data analysis and processing. The data accorded with normal distribution and homogeneity of variance, and presented using the mean ± standard deviation. Differences between the two groups were compared using independent sample *t*-tests. Count data are expressed as the rate (%), and the *χ* ^2^ test was applied for comparisons. In all analyses, *p* < 0.05 was taken to indicate statistical significance.

## Results

### 1. Basic characteristics of the patients

**Figure 1:**
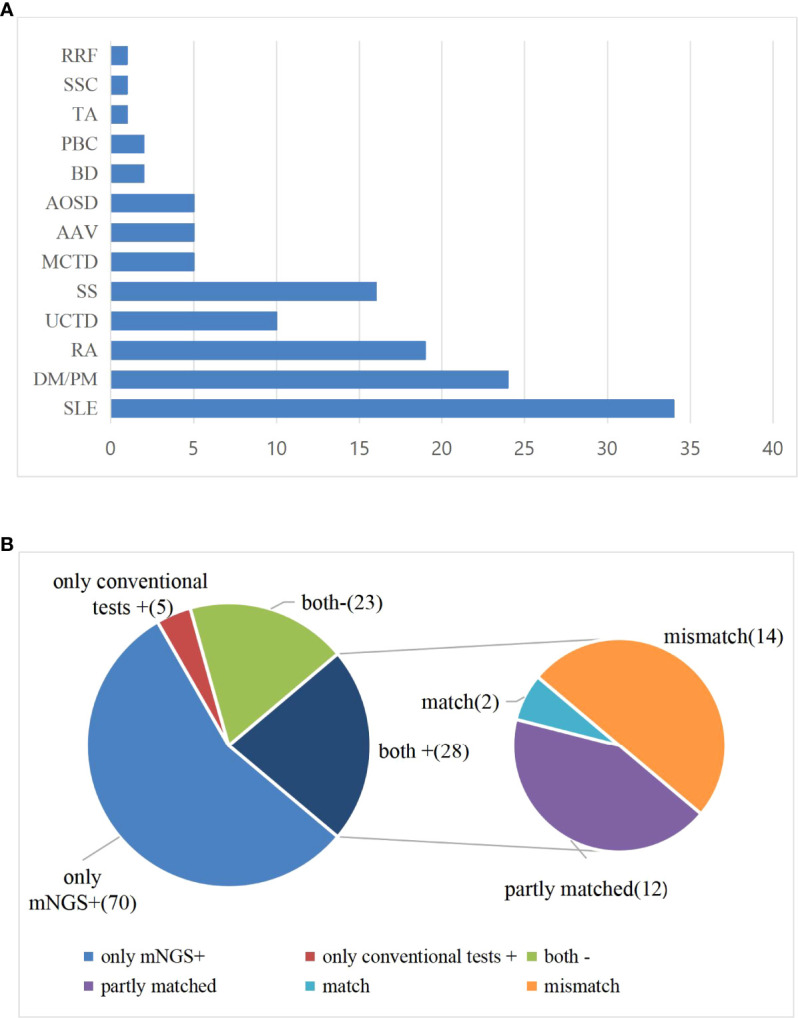
**(A)** Distribution of disease types among all enrolled patients. **(B)** Comparison of test results between mNGS and conventional diagnostic testing methods (CDT) in peripheral blood of patients with CTD. Both+, results of mNGS and CDT were both positive; both-, results of mNGS and CDT were both negative; only mNGS+, only the mNGS result was positive; only conventional testing+, only the CDT result was positive. Match, results of mNGS were totally identical with those of CDT; partly matched, results of mNGS and CDT were totally matched, mNGS often identified more pathogens than conventional methods; mismatch, results of mNGS and CDT were totally mismatched.

Among the 126 suspected infection patients with CTD, 28 patients were negative for blood mNGS, and pathogens were detected in the blood of 98 patients. Among 98 mNGS-positive patients, 46 patients were initially diagnosed with infection based on mNGS results, and the remaining 52 cases were identified as having one or more pathogen infections, which is consistent with the clinical characteristics by two physicians who specialize in the management of infection in rheumatic diseases based on their medical history, clinical manifestations, and supplementary examinations. Female patients had a high discrepancy, whether from the bloodstream infection or the non-infection group (**Table 1**). Mean age in the positive and negative group was 52.23 ± 16.72 *vs*. 50.00 ± 18.55 years, respectively. The basic laboratory data of the two groups are shown in **Table 1**. There were no significant differences in blood routine, ESR, CRP, and PCT between two groups (*p* > 0.05). SLE, DM/PM, and RA were the most common among all included suspected patients (**Figure 1A**).

**Table 1:** Baseline characteristics of all patients.

Characteristics and laboratory parameters	All patients	Positive*n = 103	Negative *n* = 23	*p*-value
**Age, mean (range), years**	50.30 ± 16.55	52.23 ± 16.72	50.00 ± 18.55	>0.05
**Sex, female, *n* (%)**	99 (78.6)	79 (73.7)	20 (87.0)	>0.05
**WBC (**×**10^9^/L)**	8.60 ± 5.22	8.86 ± 5.21	8.23 ± 6.60	>0.05
**Hb (**×**10^9^/L)**	112.52 ± 22.56	114.74 ± 22.66	111.38 ± 23.95	>0.05
**PLT (**×**10^9^/L)**	228.02 ± 114.00	223.21 ± 120.93	213.31 ± 89.26	>0.05
**Neutrophil (**×**10^9^/L)**	7.64 ± 7.57	8.10 ± 9.01	8.44 ± 7.50	>0.05
**Neutrophil%**	75.45 ± 16.96	75.05 ± 18.86	73.21 ± 24.09	>0.05
**Lymphocyte (**×**10^9^/L)**	1.09 ± 0.74	1.12 ± 0.83	1.01 ± 0.93	>0.05
**Lymphocyte%**	15.97 ± 11.24	15.29 ± 10.53	15.27 ± 9.66	>0.05
**Monocyte (**×**10^9^/L)**	1.35 ± 10.11	0.44 ± 0.25	0.40 ± 0.30	>0.05
**Monocyte%**	75.45 ± 16.97	5.80 ± 3.60	5.38 ± 2.48	>0.05
**Erythrocyte sedimentation rate (mm/h)**	64.47 ± 43.36	67.09 ± 42.34	53.92 ± 38.99	>0.05
**C-reactive protein**	53.50 ± 67.32	61.70 ± 77.87	31.82 ± 44.68	>0.05
**Procalcitonin**	2.08 ± 7.04	2.41 ± 7.81	0.56 ± 0.21	>0.05
**Glucocorticoid use, *n* (%)**	53	40 (38.8%)	13 (56.5%)	>0.05
**DMARDs use, *n* (%)**	45	33 (32.0%)	12 (52.2%)	>0.05

*The infection group included 98 mNGS positive and 5 only CDT positive. The non-infection group means negative for mNGS and CDT.

### 2. Clinical features and complications

In our cohort, 84 patients had fever, namely, 70 cases in the positive group and 14 cases in the negative group. Three of the positive patients developed infectious shock, and five had secondary hemophilic cell syndrome. Due to the immunological disorders, complex conditions, and therapeutic factors of CTD patients, multipathogen or multiple site infections often occur. The incidence of fever in the positive group and the negative group was 67.96% and 60.87%, respectively. The occurrence of pulmonary infection is higher in positive patients than in negative patients. Pulmonary infection was the most common type of co-infections in the positive group (31, 30.10%), followed by skin infections (7, 6.8%), urinary tract infection (3, 2.91%), and intestinal infections (4, 3.88%); cholecystitis, pancreatitis, encephalitis, and esophagitis also happen occasionally (**Table 2**).

**Table 2:** Clinical features and complications of all patients.

Clinical features and complications	Positive*n = 103	Negative *n* = 23	*p*-value
**Fever**	70 (67.96%)	14 (60.87%)	0.514
**Pulmonary infection**	31 (30.10%)	1 (4.35%)	0.009
**Skin infections**	7 (6.8%)	–	–
**Urinary tract infection**	3 (2.91%)	2 (8.70%)	0.199
**Intestinal infections**	4 (3.88%)	1 (4.35%)	0.918
**Acute cholecystitis**	1 (0.97%)	–	–
**Acute pancreatitis**	1 (0.97%)	–	–
**Encephalitis**	2 (1.94%)	–	–
**Fungal esophagitis**	1 (0.97%)	–	–
**Pericardial effusion**	9 (1.94%)	1 (4.35%)	0.481
**Pleural effusion**	8 (8.74%)	2 (8.70%)	0.882
**Hemophagocytic syndrome**	5 (4.85%)	–	–
**Infectious shock**	3 (2.91%)	–	–

*Positive included 98 mNGS positive and 5 only CDT positive. Negative means negative for mNGS and CDT.

### 3. Comparison of test results between mNGS and conventional diagnostic testing methods in positive CTD patients

The positive pathogen detection rates of blood mNGS and CDT were 98/126 (77.8%) and 33/126 (26.2%), respectively. The mNGS and CDT were both positive for pathogen detection in 28 individuals. Of both positive individuals, 2 cases were perfect matches, 12 cases were partly matched, and 14 cases were totally mismatched. More pathogens were identified by mNGS than by CDT in partially matched individuals. In 14 cases from whom different microbes were detected by CDT and mNGS, CDT tested positive, namely, Human gammaherpesvirus 4 (*n* = 2), *Mycoplasma pneumoniae* (*n* = 5), influenza virus B (*n* = 2), Parainfluenza virus (*n* = 1), Escherichia coli (*n* = 1),, *Enterococcus faecalis* (*n* = 1), *Legionella pneumophila* (*n* = 1), and *Staphylococcus epidermidis* (*n* = 1). mNGS identified pathogens, including Human betaherpesvirus 5 (*n* = 3), *Candida parapsilosis* (*n* = 1), *Aspergillus glaucus* (*n* = 1), *L. pneumophila* (*n* = 1), *Blastocystis hominis* (*n* = 1), Human gammaherpesvirus 1 (*n* = 1), Human gammaherpesvirus 4 (*n* = 2), *Enterobacter cloacae* (*n* = 1), *S. epidermidis* (*n* = 1), *Staphylococcus hominis*, *E. faecalis*, *Leuconostoc lactis* (*n* = 1), and Human betaherpesvirus 5 and Torque teno virus (*n* = 1).

A total of 23 cases were negative for both mNGS and CDT. Seventy cases were positive for mNGS only. There were only five cases positive for pathogen detection by CDT only (**Figure 1B**). In five CDT positive patients, two cases had influenza A virus and influenza virus B, one case had parainfluenza virus positive, and two cases were positive in the serological test of *M. pneumoniae*.

### 4. Distribution of identified pathogens in CTD patients by blood mNGS

**Figure 2:**
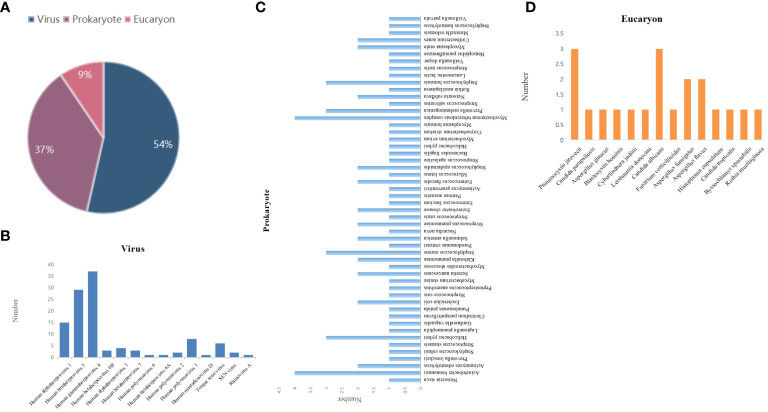
Distribution of pathogens detected by mNGS. **(A)** Type distribution of pathogens identified by mNGS. Species distribution of **(B)** viruses, **(C)** prokaryotes, and **(D)** eukaryotes detected by mNGS.

The results of mNGS showed that 131 were virus-positive (54%); 78 were prokaryote-positive (37%), including bacteria and mycoplasma; and 14 were eukaryote-positive (9%) (**Figure 2A**). According to the aggregated mNGS results, viruses were the most common pathogens identified, followed by prokaryotes and eukaryotes. Notably, these showed that the detection rate of viruses was the highest. The most common type is Human gammaherpesvirus 4 (EBV), followed by Human betaherpesvirus 5 (CMV), and Human alphaherpesvirus 1 and other types (**Figure 2B**). The most frequently detected prokaryotes were *Acinetobacter baumannii* and *Mycobacterium tuberculosis* complex, followed by *Staphylococcus aureus*, *Prevotella melaninogenica*, *Staphylococcus homini*, and *Helicobacter pylori* (**Figure 2C**). The major pathogens were *Pneumocystis jirovecii* and *Candida albicans* among eukaryote-positive individuals (**Figure 2D**). *Aspergillus fumigatus* and *Aspergillus flavus* also account for a certain percentage (**Figure 2D**).

### 5. Distribution of identified mixed infection types in CTD patients by blood mNGS

**Figure 3:**
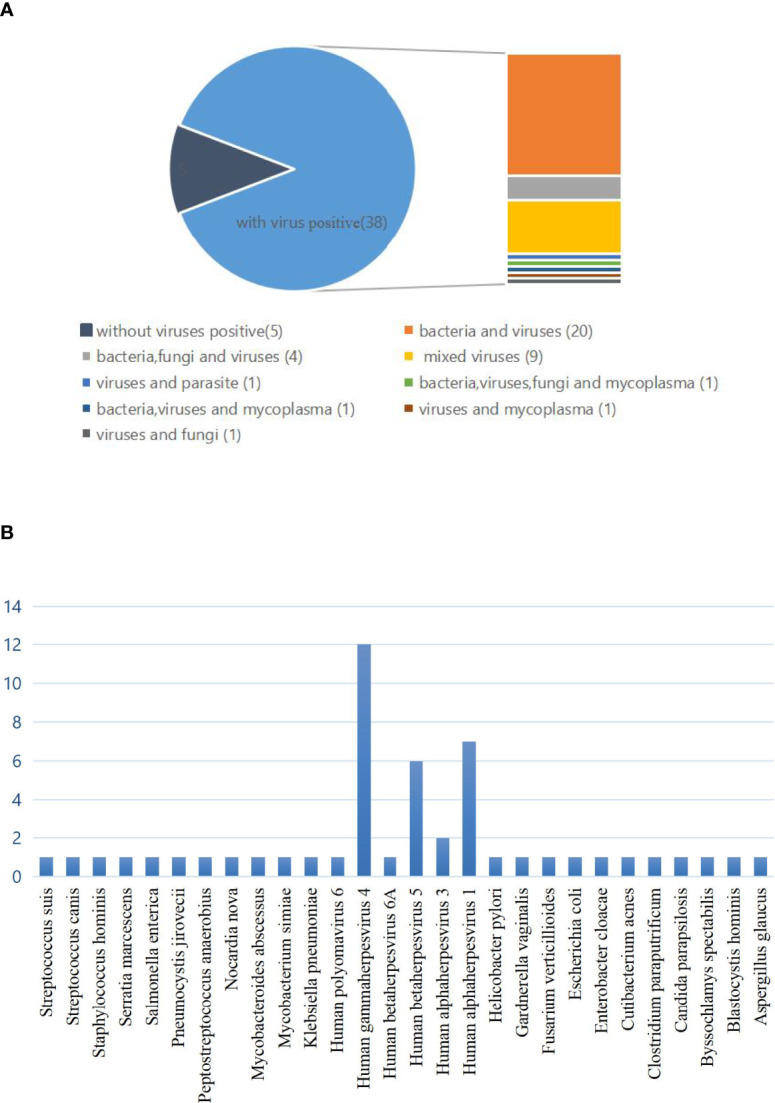
**(A)** The classification of mixed infections with or without viruses detected by mNGS and conventional diagnostic testing methods (CDT). In the mixed infection, there were 5 cases with no virus infection and 38 cases with virus infection, namely, 20 cases of bacteria and viruses infection; 4 cases of bacteria, fungi, and virus infection; 9 cases of virus mixed infection; 1 case of bacteria, virus, fungi, and mycoplasma infection; 1 case of bacteria, virus, and mycoplasma infection; 1 case of virus and mycoplasma infection, and 1 case of virus and fungi infection. **(B)** Distribution of pathogens for single infections.

The high incidence of mixed infections cannot be ignored. In the mixed infection, 5 cases were virus-negative and 38 cases were virus-positive, namely, 20 cases of bacteria and virus infections; 4 cases of bacteria, fungus, and virus infection; 9 cases of virus mixed infection; 1 case of virus and parasite infection 1 case of bacteria, virus, fungus, and mycoplasma infection; 1 case of bacteria, virus, and mycoplasma infection; 1 case of virus and mycoplasma infection; and 1 case of virus and fungus infection (**Figure 3A**). We have to admit that we cannot accurately distinguish the pathogenicity of some patients with multiple pathogens, but there is at least one or more pathogen infection according to clinical judgment. In addition, it is noteworthy that the highest detection rate in single positive cases was also virus, the most common of which was Human gammaherpesvirus 4 (EBV), followed by Human betaherpesvirus 5 (CMV) and Human alphaherpesvirus 1 (**Figure 3B**).

## Discussion

Infection is the main cause of death in CTD patients. Accurate identification of infection in CTD patients is difficult due to the impaired immune system function and the atypical or complex mixed pathogen types. In addition, the infection-related clinical manifestations and imaging features may be atypical in these kinds of patients. Therefore, it may be difficult to identify the target pathogen using conventional culture, PCR, immunofluorescence analysis, and other conventional testing methods. As an effective and rapid pathogen detection method, mNGS is necessary for the diagnosis of infection.

In our study, we compared test results between blood mNGS and CDT in all CTD patients highly suspected of infection and found that 98 cases were positive in mNGS and 33 cases were positive in CDT, namely, 28 cases both mNGS and CDT positive and 5 cases only CDT positive. In some positive individuals, more pathogens and mixed infection were identified by mNGS than by CDT. These results indicated that mNGS showed a higher sensitivity to the detection of pathogens in CTD patients, which contributed to the diagnosis of rare pathogens and mixed infection. It suggested that mNGS may be a useful tool for monitoring infection occurrence in CTD. However, in our results, two cases with influenza A virus and influenza virus B, one case with parainfluenza virus positive, two cases were positive in serological test of *M. pneumoniae* among 5 only CDT-positive patients. The above mNGS missed pathogens suggested that mNGS has certain limitations in the detection of mycoplasma and influenza viruses. In fact, rapid and high-throughput sequencing of influenza viruses remains a challenge because of the sequence diversity and evolutionary dynamics of influenza viruses (10–12). There are ongoing studies of highly sensitive and robust method improvements for influenza viruses, and future applications of new strategies could improve mNGS detection of them (11). For *M. pneumoniae*, we consider that more types of samples, such as bronchoalveolar lavage fluid (BALF), sputum, and pleural effusion, or pathogen target NGS (ptNGS) may be beneficial (13).

Although mNGS has the capacity to detect pathogens that are unidentifiable by CDT, its diagnostic performance also has limitations. In our results, of the 28 patients who were positive for both NGS and CDT, 14 cases were totally mismatched. *M. pneumoniae* and influenza virus B were mainly detected by CDT. mNGS consisted largely of Human gammaherpesvirus 4 and Human betaherpesvirus 5, and beyond that, some rare pathogens such as *A. glaucus* and *B. hominis* have also been reported. Overall, mNGS may have lower sensitivity than CDT in the detection of certain pathogens, such as influenza. However, what is undeniable is that it is a good supplement to the current pathogen detection methods, especially some pathogens that are rare and are undetectable using conventional methods.

More recently, opportunistic infections have also been increasingly reported in CTD, especially SLE and DM/PM (14–16) SLE, and DM/PM had the highest incidence of infections in our results. DM/PM and SLE are “high risk” rheumatic diseases and prone to various complications including infection. On the one hand, the continued use of immunosuppressive medications and corticosteroids will impair protective immunity, thus greatly increasing the risk of opportunistic infections. On the other hand, infection will trigger autoimmunity, aggravate immune imbalance, initiate immune inflammatory cascade, and eventually lead to the injury of host tissues and organs. In fact, there are a great number of pathogens responsible for opportunistic infections in CTD, and research has highlighted the high incidence of fungi commonly observed in DM/PM, *C. albicans*, *Pneumocystis carinii*, and *A. fumigatus*; cytomegalovirus and herpes simplex virus also account for a certain proportion (17). An outpatient study was performed in France that identified an array of opportunistic pathogens, including *C. albicans*, *P. jirovecii*, and *A. fumigatus (* 18). Similarly, in our study, *P. jirovecii* and *C. albicans* were the major pathogens among fungal infections; in addition to this, *A. fumigatus* and *A. flavus* also account for a certain percentage. The incidence of *P. jirovecii* in CTD is uncommon and quite difficult to diagnose. However, autoimmune rheumatic diseases significantly increased the overall risk of *P. jirovecii* infection compared to healthy individuals, especially SLE and DM/PM (19, 20); it portends high mortality, yet is a largely preventable complication of rheumatic disease treatment (21, 22). Still, it remains a grave concern in CTD patients due to its high mortality rate (22–24). Therefore, timely diagnosis and necessary prophylaxis are crucial for this kind of infection. Candidiasis has been reported to be one of the most common opportunistic pathogens in CTD (14, 18). However mNGS is a promising method for the rapid and accurate detection of such infections.

Furthermore, our results suggested the highest detection of viruses, although many are opportunistic pathogens. In fact, opportunistic pathogens with lower virulence in a healthy host cause more severe and frequent disease in autoimmune disorder individuals, leading to the occurrence of opportunistic infections. We summarized the identified virus types, and the results suggested that the common types of infections in CTD patients regardless of single or mixed infection were EBV, CMV, and Human alphaherpesvirus 1. The incidence of prokaryote and eukaryote infections is secondary to viruses. Meanwhile, in the mixed infection, bacteria and virus mixed infections are especially given priority. Therefore, the high detection rate of the viruses suggested that we should pay attention to the existence of viremia in CTD patients. As the most common type of virus infections, the associations of CMV and EBV infection with CTD have long been a matter of debate (25–28). EBV, also referred to as human gammaherpesvirus 4, is a double-stranded DNA (dsDNA) virus in the family Herpesviridae (29). CMV referred to human gammaherpesvirus 4, also a β-subgroup of the herpesvirus family (28). EBV- or CMV-induced autoimmunity is thought to be involved in the pathogenesis of some CTD, such as SLE and DM/PM (25, 30, 31). Huang et al. reported that CMV infections were significantly higher in idiopathic inflammatory myopathy patients, particularly in MDA5+ DM patients, and suggested that CMV might participate in the pathogenesis of MDA5+ DM by decreasing CD4+ T cells and CD19+ B cells (4). Our previous studies have also reported the high incidence of EBV and CMV infections, and found that the Th17 and Treg levels were decreased in the SLE patients with EBV or CMV viremia (32). In short, accumulating lines of evidence indicated that virus infections play important roles in CTD regardless of disease occurrence, infection progression, and prognosis. It is necessary to screen patients for CMV or EBV infections of CTD patients in clinical work. Timely antiviral therapy may help to improve prognoses.

With the application of mNGS, more pathogen types have been detected, especially opportunistic pathogens (33). In this case, interpreting mNGS results accurately and identifying opportunistic pathogens in the corresponding patients remain difficult to achieve (34). In the present study, a number of opportunistic pathogenic microorganisms were identified by blood mNGS, but these positive results posed interpretational challenges; partial results should be interpreted within the context of its limitations. The immune function of patients with CTD is often disturbed or impaired, so many opportunistic pathogens can also cause more severe and frequent disease. The causative pathogens should be cautiously differentiated based on mNGS in conjunction with clinical professional assessment and the state of immune function for guiding antimicrobial therapy for CTD patients.

There are some limitations in this study, including the fact that some patients underwent antimicrobial therapy prior to pathogen testing and all patients were drawn from one medical center, so there is potential for bias. Moreover, it is often unclear whether microbes detected using blood mGNS are contaminants, colonizers, or pathogens, which need further study especially in CTD patients with immune disorder. In particular, there may be some false positives in patients with mixed infections where multiple pathogens are detected, and how to prepare for identification of pathogens is a challenge. Finally, we only performed blood, cerebrospinal fluid, and sputum mNGS, and more specimens should be included to obtain more accurate test results.

In summary, mNGS can improve the pathogen detection and disease management of CTD patients. It has a high sensitivity and a wide detection range for microorganisms. Furthermore, the high incidence of opportunistic virus infections in CTD patients should be of sufficient concern. In some highly suspected CTD patients with severe infections or in the context of opportunistic pathogens and mixed infections, blood mNGS can be used as a supplement to conventional microbiological tests.

## Data availability statement

The data presented in the study are deposited in National Genomics Data Center (http://ngdc.cncb.ac.cn), accession number PRJCA010481.

## Ethics statement

This study was reviewed and approved by Ethics Committee of the Second Hospital of Shanxi Medical University. Written informed consent for participation was not required for this study in accordance with the national legislation and the institutional requirements.

## Author contributions

All authors were involved in drafting the article or revising it critically for important intellectual content, and all authors approved the final version to be published. Concept and design: CW, XL, and CG. Acquisition of data: RS, HY, NL, and TD. Analysis and interpretation: RS, BL, and YX. Drafting the manuscript: RS. All authors contributed to the article and approved the submitted version.

## Funding

This work was supported by the National Natural Science Foundation of China (No. 81971543 and No. 81471618) and the Key Research and Development Projects of Shanxi Province (201803D31119).

## Conflict of interest

The authors declare that the research was conducted in the absence of any commercial or financial relationships that could be construed as a potential conflict of interest.

## Publisher’s note

All claims expressed in this article are solely those of the authors and do not necessarily represent those of their affiliated organizations, or those of the publisher, the editors and the reviewers. Any product that may be evaluated in this article, or claim that may be made by its manufacturer, is not guaranteed or endorsed by the publisher.
